# Fear of COVID and Physical Health Among People Living with HIV in China: Mediation Effects of HIV Stigma, Social Support, and Substance Use

**DOI:** 10.1007/s10461-023-04099-9

**Published:** 2023-06-07

**Authors:** Wei-Ti Chen, Feifei Huang, Wenxiu Sun, Lin Zhang

**Affiliations:** 1 School of Nursing, University of California, Los Angeles, Los Angeles, CA 90095 USA; 2 School of Nursing, Fujian Medical University, Fuzhou, China; 3 Shanghai Public Health Clinical Center, Fudan University, Shanghai, CA 201500 China

**Keywords:** COVID-19 fear, Physical health, HIV stigma, Social support, Substance use, China

## Abstract

The COVID-19 pandemic has uniquely impacted people living with HIV (PLWH) worldwide. The negative impacts on PLWH’s mental health from fear of COVID-19 are labeled as “a double stress.” The association between fear of COVID-19 and HIV (internalized) stigma has been found among PLWH. Studies that explore the relationships between fear of COVID-19 and physical health outcomes are few, especially among PLWH. In this study, we explored the relationship between fear of COVID-19 and physical health among PLWH and the mediated effects of HIV stigma, social support, and substance use. A cross-sectional online survey of PLWH (*n* = 201) from November 2021 to May 2022 was carried out in Shanghai, China. The data on socio-demographics, fear of COVID-19, physical health, HIV-related perceived stigma, social support, and substance use were gathered and analyzed by structure equation modeling (SEM). In SEM analysis, fear of COVID-19 showed a significant and indirect effect on physical health (*β*=-0.085) which was primarily mediated by HIV stigma. In SEM analysis, the final model had a good fit. Fear of COVID-19 showed a significant effect on HIV stigma (*β* = 0.223) with the majority being direct effects (*β =* 0.262) and a small indirect effect via substance use (*β=*-0.039). Furthermore, HIV stigma showed a significant effect on physical health (*β*=-0.382), the majority of which was direct (*β=*-0.340), and a small indirect effect via social support (*β=*-0.042). This is one of the first studies to explore how fear of contracting COVID-19 can affect PLWH’s coping behaviors (e.g., using substances and obtaining social support) used to combat HIV stigma as well as to achieve better physical health in China.

## Introduction

The COVID-19 pandemic has uniquely impacted people living with HIV (PLWH) worldwide, even after COVID-19 has become milder [1]. Compared with the general population, PLWH were more likely to contract COVID-19, have greater severity, and have higher death rates from COVID-19 infections, due to factors such as immune suppression, chronic inflammation, and other underlying conditions associated with HIV infection [2]. As a result, fear of contracting COVID-19 is even more prominent among PLWHs and as high as 83.3% of PLWHs reported being afraid of contracting COVID-19 [3]. Emerging evidence suggests excessive and COVID-19 fear could have a dysfunctional and damaging effect on mental health and well-being [4]. West et al., (2022) found that the negative impacts on PLWH’s mental health from fear of COVID-19 resulted in “a double stress” to the individual [5].

Studies that explore the relationships between fear of COVID-19 and physical health outcomes are few, especially among PLWH. According to Uncertainty Management Theory [6], during highly uncertain situations of the pandemic, where the status quo and security of the PLWH are challenged, the fear of COVID-19 can create a higher level of uncertainty about the future in which the uncertainty is associated with the decline of physical health [4]. Thus, it is reasonable to assume that in PLWH populations, fear of COVID-19 could contribute to poor physical health and be represented by pain interference, lowered physical function, worsened pain intensity, and diminished ability to participate in social roles and activities/satisfaction with participation in social roles [7]. However, the possible mechanism between the fear of COVID-19 and poor physical health is still unknown [7].

The association between fear of COVID-19 and HIV stigma has been found among PLWH [8]. HIV itself is a highly stigmatized condition that may generate HIV internalized stigma in PLWH - negative attitudes associated with HIV are internalized and accepted as applicable to themselves [9]. While fear of COVID-19 in PLWH may fuel their prejudicial and discriminatory attitudes toward the disease [9]. Furthermore, Rueda et al.’s (2016) meta-analysis support the notion that HIV-related stigma could have a detrimental impact on a variety of health-related outcomes in PLWH [10]. It seems reasonable, then, to expect an automatic somatization process where COVID-19 fear is related to physical health through HIV-related stigma. This stigma distress is considered an automatic reaction to fear that, without the intervention of more deliberative regulation or processing, is related to physical health problems. Thus, we propose hypothesis one (H_1_): HIV stigma mediates the relationship between COVID-19 fears and physical health problems.

PLWHs are disproportionately impacted by the fear of COVID-19 and may suffer increased psychosocial burdens as a result of the COVID-19 pandemic [10]. According to the Stress-Coping Model, when facing the double stresses of fear of COVID-19 and HIV stigma, some PLWH may apply maladaptive strategies (e.g., substance use) to manage their negative affect [3], while others may seek social support, one form of resilient coping, and defined as participation in social activities, having someone to help with physical needs, and experiencing affection, companionship and assistance, which is helpful to deal with the negative impact of stressors [11]. Thus, we propose hypothesis two (H_2_): the sequential mediation of substance use, HIV stigma, and social support between fear of COVID-19 and physical health problems.

Additionally, it is important to consider the unpredictable and uncertain nature of the pandemic and related issues, such as lockdown periods. Taking Shanghai, China as an example, when the lockdown measures were implemented in most countries around the globe during the end of 2020, China’s policies were relaxed to cover only communities with active COVID-19 outbreaks - resulting in community-to-community lockdowns [12]. However, inter-city and -province travel was allowed, with a series of negative polymerase chain reaction (PCR) tests done within 24 h of travel and a “green light” sent via the cellphone number associated with the travelers. From February to August 2022, a second wave of Omicron variants erupted and lockdown measures were once again implemented in Shanghai and included the closure of schools, restrictions on public gatherings, a country-wide curfew, stay-at-home-orders, restricted local travel, and a total travel ban in and out of the country. Compared with the general population, PLWHs experienced greater difficulties and challenges due to lockdowns such as reduced assess to HIV care services and difficulties in accessing nutrition and cleaning products, which may have been detrimental to the health and well-being of PLWHs [13].

**Fig. 1:**
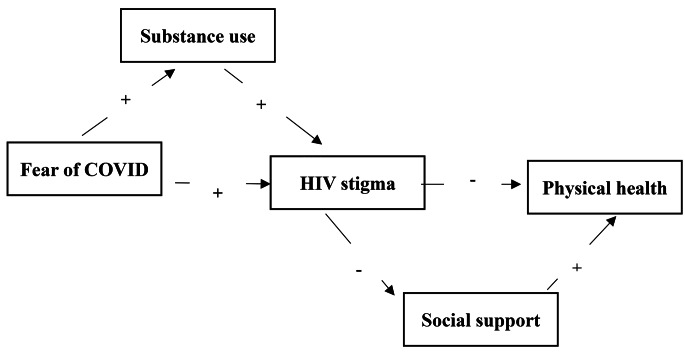
Hypothesized relationships model

Based on a recent literature review, there remain gaps in detecting and comparing the mediation effect of HIV internalized stigma (as during lock down, there was no interaction with outside society), social support, and substance use between fear of COVID-19 and physical health outcomes, forming an impetus for this study. By closing these informational gaps, the study sheds light on the potential negative psychological effects of the COVID-19 pandemic on the physical health of PLWH, which informs public health policies aimed at improving the physical health and well-being of PLWH during and after and COVID-19 pandemic. On the other hand, understanding the mediating effects of substance use and social support is fundamental to the development of more targeted interventions for mitigating fear of COVID-19 and to further promote better physical health outcomes among PLWHs. We propose two hypotheses (see Fig. 1): (1) H_1_: HIV stigma mediates the relationship between fear of COVID-19 and physical health; (2) H_2_: substance use, HIV stigma, and social support sequentially mediate the relationship between fear of COVID-19 and physical health problems.

## Method

### Sample, Setting, and Procedure

This is a cross-sectional online survey and structural equation modeling analysis study. A total of 201 of PLWH were recruited from November 2021 to May 2022 in Shanghai, China. The relevant institutional ethical review boards approved the study. The study conformed with the Strengthening the Reporting of Observational Studies in Epidemiology (STROBE) statement [14].The study inclusion criteria were as follows: (a) confirmed HIV serostatus, (b) willing to participate in the survey, and (c) at least 18 years old. Patients were excluded from the study if they were unable to complete the study survey in Chinese or had a severe impairment in their physical or mental functions. If they agreed to participate and were able to provide informed consent, an individualized survey link was sent to them via WeChat (a free cell phone application similar to text messaging). After completing the survey, participants were reimbursed for their participation and time.

We used an a priori sample size calculator for Standard Error of Mean (SEM; which is specially designed for calculating the sample size of SEM [15]. The minimum sample size with a moderate effect (0.3), at a power value of 0.9, including 5 latent and 5 observed variables, and with a value of 0.05 was calculated as 188. Hence, we chose a minimum sample size of 188.

### Measures

Each participant completed a set of standardized measures using the Chinese online survey platform, Wenjuanxing (aka Questionnaire Star (QS), which is similar to Survey Monkey). Data gathered included socio-demographics, fear of COVID-19, physical health, HIV-related perceived stigma, social support, and substance use. The survey took 30–45 min to complete. The survey measures have been tested in PLHW populations and have shown strong reliability and validity over time.

#### Socio-demographic Characteristics

Participant age, gender, place of residence, educational level, current working status, years of living with HIV, antiretroviral therapy (ART) use, and recent viral load measures were collected.

#### The Fear of COVID-19 Scale

The seven-item unidimensional scale was used to measure fear of COVID-19 among the general population. Each item was measured on a 5-point Likert scale (1 = strongly disagree to 5 = strongly agree). The higher the total score, the greater the fear of COVID-19. For this scale, the overall Cronbach’s alpha coefficient (ἀ) was 0.82 [16] and 0.91 for this study.

#### The Patient-Reported Outcomes Measurement Information System (PROMIS) Global-10

The PROMIS Global-10 is a multi-dimensional 10-item measure of general health, across conditions of physical functioning, anxiety, depression, fatigue, sleep disturbance, satisfaction with participation in social roles, and pain intensity. Each item was measured with ranges from 1 to 5 (i.e., 16 decrements each), except a single item in pain intensity that was assessed using a single 11-point numeric rating scale anchored between no pain (0) and worse imaginable pain (10). Specific components of the PROMIS Global-10 correlate with physical health outcomes and others correlate with mental health, yielding a total physical health score and a total mental health score. Sample questions in physical health include, “how would you rate your fatigue on average?” and “how would you rate your pain on average?”. In this study, we only used the physical health score. Raw PROMIS-10 scores for physical health were calculated and converted to standardized T-score values. T-scores have a mean of 50 and a standard deviation of 10 in the general population, and higher scores indicate better physical health. The Cronbach’s alpha coefficient (ἀ) ranged from 0.87 to 0.97 among 1,306 PLWHs in the United States [17] and 0.92 for this study.

#### The Medical Outcomes Study–Social Support Survey (MOS-SSS)

The 6-item 1-factor MOS-SSS was used to measure social support, which was adapted from the 19-item MOS-SSS [18]. All items are rated using a 5-point Likert scale (1 = “*none of the time*” to 5 = “*all of the time*”). A higher score indicated a higher level of social support. The overall Cronbach’s alpha coefficient (ἀ) for this sample was 0.87.

#### A Brief Measure of Stigma for HIV + Youth

The stigma instrument for HIV + Youth [19] was adapted from the 40-item Berger’s HIV stigma scale [20]. All of the items are rated using a 4-point Likert scale (1 = “strongly agree” to 4 = “strongly disagree”). These questions were selected and tested with a focus on internalized stigma [19]. A higher score indicated a higher level of HIV internalized stigma. The overall Cronbach’s alpha coefficient (ἀ) for this sample was 0.89.

#### The Alcohol, Smoking, and Substance Involvement Screening Test (ASSIST)

The 10-item ASSIST [21] was used to measure the frequency of substance use. Substance use referred to the non-medical use of any substance falling into 10 categories over the past 3 months before the study. The instrument included frequencies of tobacco products, alcoholic beverages, cannabis, cocaine, stimulants, inhalants, sedatives/hypnotics, hallucinogens, opioids, and other drug use. The overall Cronbach’s alpha coefficient (ἀ) for this sample was 0.77.

### Data Analysis

We conducted data analyses using SPSS 24.0 and AMOS 23.0 (IBM, Chicago, IL). The continuous variables were expressed as means and standard deviations (SD). Categorical variables were expressed as proportions or percentages. We calculated whether the participant groups before a before lockdown and during a lockdown were different by Chi-Square (including Fisher’s exact test). First, we conducted Pearson’s correlation analyses to examine the relationships among fear of COVID-19, physical health, social support, HIV stigma, and substance use. Second, we used the bias-corrected percentile bootstrap method (repeated 5,000 times) to test multiple mediation analyses to explore the mechanism through which fear of COVID-19 can be influenced by physical health when considering social support, HIV stigma, and substance use as mediators. The following fit indices of the model were used [21]: normed chi-square (χ2/df, 1.0~3.0, *p* > 0.05), root mean square error of approximation (RMSEA < 0.08), comparative fit index (CFI > 0.9), and Tucker-Lewis Index (TLI, > 0.9).

## Results

### Sample Characteristics

Among the total sample of 201 PLWHs, 98.5% were male (*n* = 198), with a mean age of 39.79 years (*SD* = 11.11, range = 18–67) and the average years of living with HIV was 6.77 years (*SD* = 4.90). More than three quarters (87.1%, n = 175) of PLWH’s recent viral load was undetectable. Only 16.2% (*n* = 32) of PLWH had other disease in addition to HIV. The details of the socio-demographic characteristics of the participants are presented in Table 1. The data demonstrate that there are no significant differences in the demographic characteristics of the two participant groups that were surveyed before and during the lockdown periods.

**Table 1:** Socio-demographic characteristics of participants (*n* = 201)

Variables	*Beofre Lockdown n* (%)	*During Lockdown n* (%)	*P-Value*
Gender			0.577^a^
Male	114 (99.1)	84 (97.7)	
Female	1 (0.9)	2 (2.3)	
Place of residence			0.912 ^a^
City	105 (91.3)	78 (90.7)	
Township	8 (7.0)	7 (8.1)	
Rural	2 (1.7)	1 (1.2)	
Educational level			0.166 ^a^
Primary school or below	1 (0.9)	4 (4.8)	
Junior high school	7 (6.1)	7 (8.3)	
Senior high school graduation	15 (13.2)	17 (20.2)	
Professional (vocational) training school or some college	27 (23.7)	13 (15.5)	
Bachelor’s degree or above	64 (56.1)	43 (51.2)	
Current working status			0.501 ^a^
Working	83 (72.2)	59 (68.6)	
Student	1 (0.9)	2 (2.3)	
Retired	10 (8.7)	12 (14.0)	
Not working	21 (18.3)	13 (15.1)	
Currently using ART			0.393 ^a^
Yes	110 (95.7)	85 (98.8)	
No	4 (3.5)	1 (1.2)	
Recent viral load			0.033*
Undetectable	99 (86.1)	76 (88.4)	
Detectable	13 (11.3)	3 (3.5)	
Unknown	3 (2.6)	7 (8.1)	
Have other diseases			0.129
Yes	90 (80.4)	76 (88.4)	
No	22 (19.6)	10 (11.6)	

a: Fisher’s exact test; ^*^p < 0.05

### Bivariate Correlations

As shown in Table 2, physical health was significantly negatively correlated with fear of COVID-19 and HIV stigma, while positively correlated with social support. Fear of COVID-19 was significantly positively correlated with HIV stigma and substance use. Furthermore, HIV stigma was significantly negatively correlated with substance use but positively correlated with social support.

**Table 2:** Descriptive statistics and bivariate correlations

	Mean (SD)	1. (FC)	2 (HS)	3. (SS)	4. (SU)	5. (PH)
1. Fear of COVID (FC)	17.93 (7.06)	-				
2. HIV stigma (HS)	33.11 (8.16)	0.227**	-			
3. Social support (SS)	12.35 (4.32)	− 0.135	− 0.268**	-		
4. Substance use (SU)	3.27 (3.86)	− 0.197**	0.166*	0.041	-	
5. Physical health (PH)	14.34 (2.69)	− 0.251**	− 0.374**	0.318**	− 0.138	-

*Notes.* SD = standard deviation; ^*^p < 0.05; ^**^p < 0.01

### Structural Equation Model

**Fig. 2:**
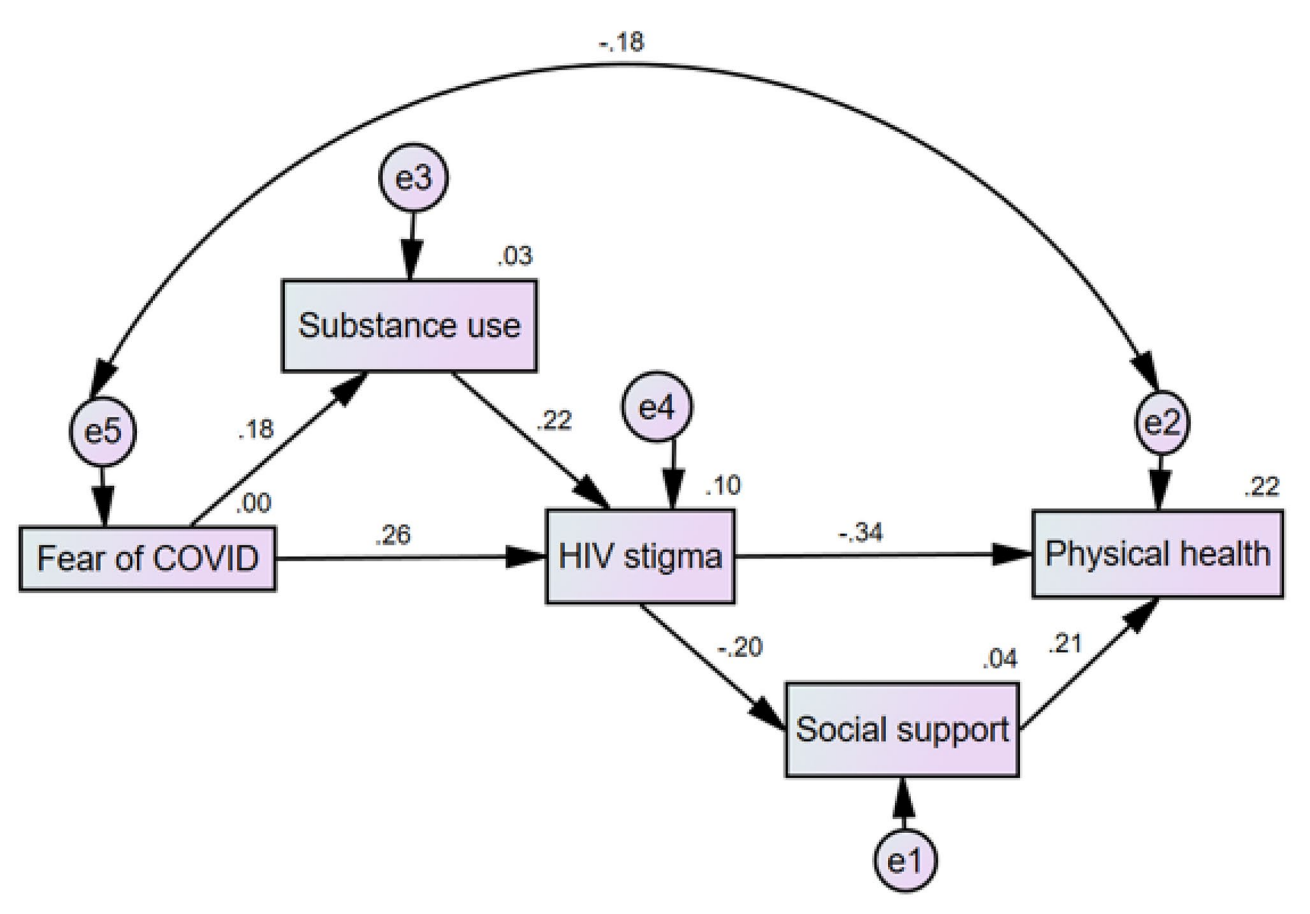
Final structural equation model

The structural equation model was initially tested by controlling for socio-demographic variables. According to the initial findings, we correlated the error of fear of COVID and physical health due to the modification indices being bigger than 15. The final model fit the data adequately and satisfied our preset criteria (Fig. 2), χ2/df = 1.585 (*p* = 0.191), RMSEA = 0.054, CFI = 0.98, NFI = 0.951. All paths showed a significant statistical effect (*p* < 0.05). The effect of the values of each variable on fear of COVID-19 is summarized in Table 3.

**Table 3:** Standardized estimates of direct and indirect effects on physical health

Dependent variable	Independent variable	Direct effect	Indirect effect	Total effect
Substance use	Fear of COVID	0.177*	/	0.177*
HIV stigma	Fear of COVID	0.262*	-0.039*	0.223*
Social support	Fear of COVID	/	-0.044*	-0.044*
Physical health	Fear of COVID	/	-0.085*	-0.085*
HIV stigma	Substance use	0.224*	/	0.224*
Social support	Substance use	/	-0.044*	-0.044*
Physical health	Substance use	/	-0.085	-0.085*
Social support	HIV stigma	-0.198*	/	-0.198*
Physical health	HIV stigma	-0.340*	-0.042**	-0.382*
Physical health	Social support	0.215*	/	0.215*

^*^p < 0.01

Fear of COVID-19 showed a significant and indirect effect on physical health (effect value=-0.085) which was primarily mediated by HIV stigma. Thus, H_1_ was supported and clarified. Fear of COVID-19 showed a significant effect on HIV stigma (effect value = 0.223), the majority of which being direct effects (0.262) and a small indirect effect via substance use (-0.039). Furthermore, HIV stigma showed a significant effect on physical health (effect value=-0.382), the majority of which was direct (-0.340), and a small indirect effect via social support (-0.042). Thus, H_2_ was supported and clarified.

## Discussion

In this study, we confirmed that HIV stigma mediates the relationship between fear of COVID-19 and physical health and stigma. Also, substance use mediates fear of COVID-19 and HIV stigma. In addition, social support sequentially mediates the relationship between HIV stigma and physical health symptoms in PLWH in Shanghai, China. This is one of the first studies to explore how fear of contracting COVID-19 can affect PLWH’s coping behaviors (e.g., using substances and obtaining social support) used to combat HIV stigma as well as to achieve better physical health in China.

As the Chinese government recently revoked all travel restrictions inside and outside of China, people are reported to be contracting COVID-19 at a very fast rate [22]. PLWH are especially concerned about their compromised immunity states and their prognoses if they should become COVID-19 positive. With possible unknown variants evolving worldwide [23], PLWH are hoping to obtain support from the healthcare system but are wary of potential HIV disclosure and the stigma they may face. In our study, using substances is one way of coping with the stress and fear of contracting COVID-19. The higher the COVID-related fear, the higher the frequency of using substances, which triggered higher HIV perceived- and self-stigma.

Those PLWH who had higher fear of contracting COVID-19 reported worse physical health, which may be due to higher fatigue and pain, not only from their disrupted access to their antiretroviral therapy but also as a result of their fear of contracting COVID-19. This also provides evidence that internalized stigma mediated the uncertainty of fear of contacting COVID-19 and worsened physical health. In the SEM analysis, the higher the fear of contacting COVID-19, the worse the physical health of PLWH. Meanwhile, the higher the fear of contacting COVID-19, the higher internalized stigma. As PLWH with flu-like symptoms presented themselves in hospital, they felt they may need to disclose that they may be COVID-19 positive, as well as their HIV serostatus. Not surprisingly then, those with higher levels of internalized stigma reported worse levels of physical health. During the study’s data collection period in China, people were mandated to screen for SARS-COV-2 by PCR daily or every other day to ensure their COVID-19 status. If one member of the residential building tested COVID-19 positive, then the whole building needed to be in quarantine for 14 days [24]. Fatigue is one of the most frequently reported symptoms of PLWH in China [25]. Since many PLWH were not sure whether their fatigue was from HIV or from contracting COVID-19, they were particularly anxious during the regular screenings that they needed to both disclose their COVID-19 status and their HIV serostatus. Dual stigma is very difficult to bear as seen in this study, where PLWH who had higher fear of COVID-19 presented with worse physical health. Moreover, the higher the fear of contracting COVID-19, the higher the HIV-related stigma and worse perceived physical health.

Those who experience HIV-related stigma experience less social support. This sentiment is echoed in several previous studies [26–28] that found the higher the social support, the less the HIV-related stigma. Results from this study demonstrate that the higher the social support, the better physical health, and the higher the HIV-related stigma, the worse the physical health. Since social support can mediate physical discomfort, healthcare providers should refer PLWH during the pandemic to seek more family and social support. PLWH who are concerned about their HIV disclosure and anticipated stigma will be especially comforted by friends and family members who know their serostatus.

This early study on people living with HIV (PLWH) in China during the COVID-19 pandemic has several limitations. First, study participants experienced different restrictions within China during the data collection period. In February 2022, a lockdown order was issued in Shanghai for several months, causing inconvenience and discontent among some participants, which could have potentially skewed the data. However, this study provides one of the few datasets collected before, during, and after lockdown periods in China. Second, the resources and COVID-19 policies in Shanghai may differ from those in other rural or suburban areas in China. Therefore, the study findings cannot be generalized to other regions in China. Third, as most PLWH in China are men who have sex with men, the study participants were predominantly male, and the study results may not reflect the experiences of female PLWH in China. Fourth, the brief measure of stigma for youth living with HIV in this study was not designed for adult participants, however, the scale was a shortened version of Berger’s stigma scale which was widely used in adults living with HIV. Lastly, 16.2% (n = 32) of the study participants had other diseases in addition to HIV and may be taking medication besides antiretroviral therapy, which could affect their physical condition.

This study findings have several relevant implications for public health practice. First, the findings indicate that HIV internal stigma plays an important role in the fear of COVID-19 and physical health. Therefore, culturally sensitive evidence-based stigma-reducing interventions should be integrated into public health protocols and healthcare measures to cope with the pandemic. Specifically, people affected by the fear of COVID-19 could be involved in the development and implementation of stigma reduction interventions. In addition, to decrease both HIV-related stigma and physical discomfort in PLWHs in China, healthcare providers should pay more attention to potential substance use and encourage PLWHs to seek support from friends and families. For example, one approach could be to hold workshops or support groups specifically for PLWHs that focus on substance use and addiction. These workshops or groups could provide education on the risks associated with substance use and addiction, as well as offer strategies and coping mechanisms for managing substance use. Additionally, healthcare providers could collaborate with community organizations and integrate substance use screening and interventions into routine HIV care. Another approach could be to provide information on support groups or counseling services for PLWHs and their loved ones, as well as facilitate communication between PLWHs and their support networks.

## Conclusion

In this study, SEM is used to confirm that HIV stigma mediates the relationship between fear of COVID-19 and physical health. Results found that substance use mediates fear of COVID-19 and HIV stigma and, social support mediates the relationship between HIV stigma and physical health symptoms in PLWH in Shanghai, China. To decrease HIV-related stigma and decrease physical discomfort in PLWHs in China, healthcare providers should pay more attention to potential substance use and encourage PLWHs to seek support from friends and families. Further studies should investigate if PLWH in other parts of the world experience similar relationships between COVID-19 fear, physical health, substance use, social support, and stigma.
