# Correction: Fear of COVID and Physical Health Among People Living with HIV in China: Mediation Effects of HIV Stigma, Social Support, and Substance Use

**DOI:** 10.1007/s10461-024-04359-2

**Published:** 2024-05-09

**Authors:** Wei-Ti Chen, Feifei Huang, Wenxiu Sun, Lin Zhang

**Affiliations:** 1 School of Nursing, University of California, Los Angeles, Los Angeles, CA 90095 USA; 2 School of Nursing, Fujian Medical University, Fuzhou, China; 3 Shanghai Public Health Clinical Center, Fudan University, Shanghai, 201500 CA China

There were omissions on pages 2, and 8 of the article, as detailed below.

In the Method section (page 2), the second sentence should be added as follows: “The study design and conceptualization are partially adapted from the International Nursing Network for HIV Research study protocol (Cuca et al., 2024)”.

In the Acknowledgement section (page 8), a second and third sentences should be added as follows:

“We would like to acknowledge the entire study team from the International Nursing Network for HIV Research. The content is solely the responsibility of the authors and does not necessarily represent the official views of the National Institutes of Health, or any other funding agency.”

In the Reference section (page 8), the following citation should be included:

Cuca Y. P., Horvat Davey C., Corless I.B., Phillips J.C., Sierra-Perez A.J., Solís Báez S., Iwu E., Sabone M., Mulaudzi M.T., Murphey C., Shaibu S., Chen W-T., Santa Maria D., Schnall R., Palmieri P., Apiruknapanond P., Wang T., de Jesús T., Huang E., Broussard J., and Dawson-Rose C. (2024). The Social, Mental, and Physical Health Impacts of the COVID-19 Pandemic on People with HIV: Protocol of an Observational International Multi-Site Study. Journal of the Association of Nurses in AIDS Care, 35(1), 60-74.

